# A High‐Quality Reference Genome and Comparative Genomics of the Widely Farmed Banded Cricket ( *Gryllodes sigillatus* ) Identifies Selective Breeding Targets

**DOI:** 10.1002/ece3.71134

**Published:** 2025-03-16

**Authors:** Shangzhe Zhang, Kristin R. Duffield, Bert Foquet, Jose L. Ramirez, Ben M. Sadd, Scott K. Sakaluk, John Hunt, Nathan W. Bailey

**Affiliations:** ^1^ School of Biology University of St Andrews Fife UK; ^2^ USDA‐ARS Geospatial and Environmental Epidemiology Research Unit Mississippi State Mississippi USA; ^3^ USDA‐ARS, National Center for Agricultural Utilization Research Crop BioProtection Research Unit Peoria Illinois USA; ^4^ School of Biological Sciences Illinois State University Normal Illinois USA; ^5^ McGuire Center for Lepidoptera and Biodiversity Florida Museum of Natural History, University of Florida Gainesville Florida USA; ^6^ School of Science Western Sydney University Penrith New South Wales Australia

**Keywords:** banded cricket, cricket farming, genome‐assisted breeding, *Gryllodes sigillatus*, insect protein

## Abstract

Farmed insects have gained attention as an alternative, sustainable source of protein with a lower carbon footprint than traditional livestock. We present a high‐quality reference genome for one of the most commonly farmed insects, the banded cricket *Gryllodes sigillatus* . In addition to its agricultural importance, *G. sigillatus* is also a model in behavioural and evolutionary ecology research on reproduction and mating systems. We report comparative genomic analyses that clarify the banded cricket's evolutionary history, identify gene family expansions and contractions unique to this lineage, associate these with agriculturally important traits, and identify targets for genome‐assisted breeding efforts. The high‐quality *G. sigillatus* genome assembly plus accompanying comparative genomic analyses serve as foundational resources for both applied and basic research on insect farming and behavioural biology, enabling researchers to pinpoint trait‐associated genetic variants, unravel functional pathways governing those phenotypes, and accelerate selective breeding efforts to increase the efficacy of large‐scale insect farming operations.

## Graphical abstract

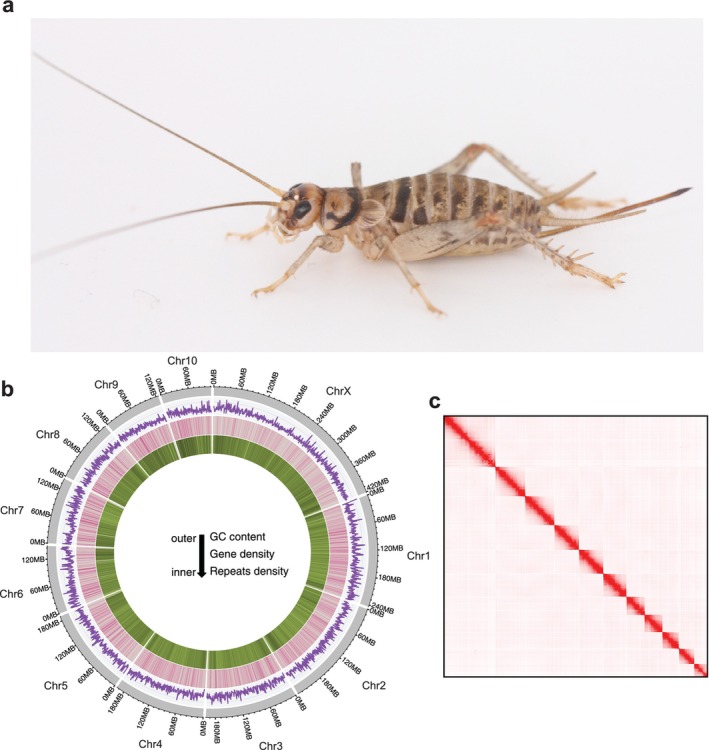

## 1 Introduction

**FIGURE 1:**
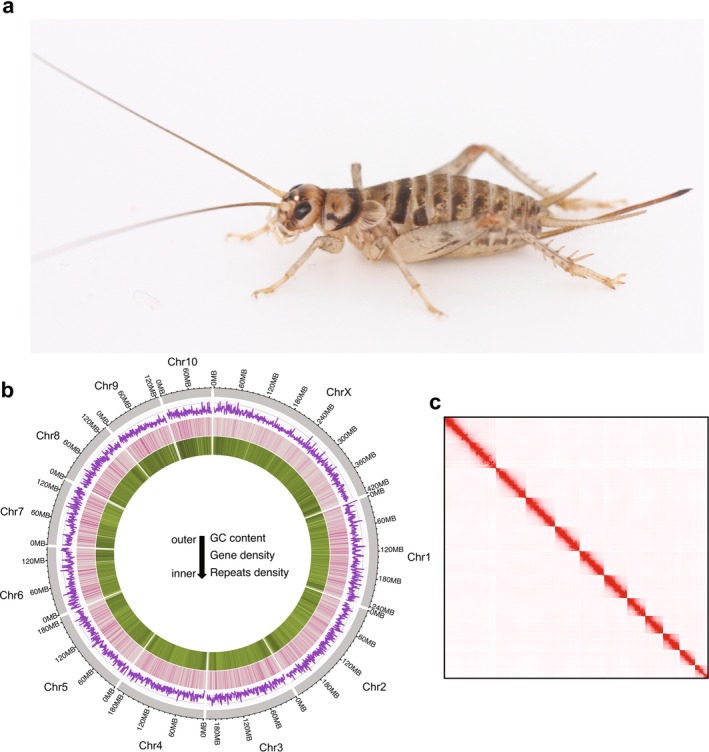
*Gryllodes sigillatus* and its genome characteristics. (a) A female *G. sigillatus* . (b) Features of the *G. sigillatus* genome. Outer track (grey) illustrates the 11 chromosomes, with the largest identified as the putative X chromosome on the basis of a published karyotype study (You et al. 2007). Genome position is indicated by the outer numbers. The inner purple track shows GC content, and the inner pink and green tracks show genome‐wide gene and repeat density, respectively. (c) Hi‐C contact map of the genome assembly indicates high contiguity and coverage. Photo credit: NW Bailey.

Global demand for sustainable protein sources has driven interest in alternative agriculture. Such efforts include insect farming, which can meet nutritional needs while significantly reducing environmental impacts (Grabowski et al. 2022; van Huis and Oonincx 2017). Insects are known for their favourable feed conversion efficiency. They require significantly less land, water, and energy resources compared to traditional livestock, exploit diverse dietary proteins (Morales‐Ramos et al. 2020; Kasdorf et al. 2025), and produce fewer greenhouse gases (Lange and Nakamura 2023). These advantages make them an environmentally friendly option for addressing food security challenges (van Huis and Oonincx 2017). Crickets (suborder Ensifera) have been farmed and consumed by humans for millennia, and over 60 species are known to be consumed across 49 countries (Magara et al. 2021). Among these, the banded cricket ( *Gryllodes sigillatus* , Figure 1a) has gained considerable attention due to its rapid growth rate, high reproductive capacity, and palatability, making it a preferred species for large‐scale farming operations (Kong et al. 2024; Magara et al. 2021).

Despite the growing commercial significance of *G. sigillatus* , publicly available genetic resources for this species are limited. This scarcity of genetic information constrains the potential for selective breeding programs that could enhance desirable traits such as nutritional value, growth rate, and reproductive efficiency. Recent research on *G. sigillatus* has identified key environmental factors that influence such traits, including temperature, food stress (Rapkin et al. 2018; Kasdorf et al. 2025; Muzzatti et al. 2025b, 2025a), mating strategies (Sakaluk et al. 2019), and pests (Muzzatti et al. 2025b). However, genome‐based selection has become central to modern breeding in a wide variety of agricultural species, resulting in rapid and efficient improvement of specific traits (Johnsson 2023). Beyond their economic importance, field crickets—including *G. sigillatus* —have long been a study organism in evolutionary and behavioural ecology (Bateman and MacFadyen 1999; Champagnon and del Cueva Castillo 2008; Sakaluk et al. 2002, 2019). Males of this polygynandrous species produce a costly nuptial gift—the spermatophylax—which they transfer to females upon mating, making *G. sigillatus* a prominent research model for examining mating system evolution, mate choice, and sexual selection (Ivy and Sakaluk 2005; Sakaluk 1984, 1991). A high‐quality genome assembly serves as a foundational resource for such efforts, enabling researchers to pinpoint trait‐associated genetic variants, unravel functional pathways governing those phenotypes, and accelerate both selective breeding efforts and fundamental research in behavioural and evolutionary biology (Sinha et al. 2021).

In this study, we produced a high‐quality, annotated, chromosome‐level reference genome for *G. sigillatus* by integrating long‐read PacBio HiFi data with high‐throughput chromosome conformation capture (Hi‐C) data. Using comparative genomic approaches, we refined the phylogenetic placement and evolutionary history of *G. sigillatus* . We identified candidate gene families linked to economically significant traits that underwent changes during the divergence of *G. sigillatus* from sister taxa and illuminated gene family expansions and contractions relevant to the unique biology of this species. This research provides a foundational resource for the genetic analysis and farming of *G. sigillatus* , offering valuable insights into its genomic features and agriculturally important traits, as well as for basic research on the evolution of mating systems and reproductive behaviour.

## 2 Methods

### 2.1 Sampling and DNA Sequencing

For whole genome sequencing, an individual *Gryllodes sigillatus* female was taken from an inbred laboratory stock line descended from approximately 500 adults collected at Las Cruces, New Mexico, in 2001 (Ivy and Sakaluk 2005). We extracted high molecular weight genomic DNA from liquid nitrogen flash‐frozen cricket legs. Following an initial wash with 1× PBS buffer, DNA was extracted using the Macherey‐Nagel NucleoBond High Molecular Weight DNA Kit. DNA quality and concentration were assessed with both Nanodrop and Qubit. Sequencing library preparation and whole genome sequencing were performed using the PacBio SMRT system by BGI Genomics, resulting in 96.25 Gb of HiFi reads with a coverage depth of approximately 48×. Additionally, flash‐frozen muscle tissue was used to prepare and sequence a Hi‐C library, also by BGI Genomics, resulting in 151.48 Gb of ca. 72× Hi‐C reads (Table S1).

### 2.2 Bulk RNA‐Seq

RNA‐seq data used to facilitate genome annotation was generated using two different methods. This was done so as to include reads from as wide a range of tissues as possible to increase the completeness of the genome annotation. We used data from whole bodies and accessory glands of males, plus whole bodies, ovaries, and heads of females (Table S2).

Whole body RNA‐seq data was generated from laboratory stock crickets as follows. These were descended from 500 adult *G. sigillatus* collected in Las Cruces, New Mexico (USA) in 2001 (Ivy and Sakaluk 2005). Rearing followed previously described methods (Duffield et al. 2021, 2022). Approximately 500 crickets were housed in ventilated, 55 L plastic storage bins packed with egg carton. Crickets were given *ad libitum* food (approximately equal parts Purina Cat Chow Complete Cat Food and Teklad Global 18% Protein Extruded Rodent Diet pellets) and water (glass vials plugged with moist cotton). Colonies were housed in an environmental chamber at 32°C on a 16:8 h light: dark cycle. Experimental crickets were frozen and stored at −80°C at 7 days post‐eclosion and shipped on dry ice to LC Sciences (Houston, TX) for further processing. Total RNA was extracted using Trizol (Invitrogen, USA) following the manufacturer's procedure. Total RNA quality and quantity were analysed using a Bioanalyzer 2100 and RNA 6000 Nano LabChip Kit (Agilent, USA), with samples obtaining RIN number > 7.0. Then, mRNA was purified in two rounds using Dynabeads Oligo (dT) (Thermo Fisher, USA) and subsequently fragmented with a Magnesium RNA Fragmentation Module (New England Biolabs, USA) at 94°C for 5‐7 min. The cleaved RNA fragments were reverse‐transcribed to cDNA using SuperScript II Reverse Transcriptase (Invitrogen, USA) and then used to synthesise U‐labelled second‐stranded DNAs with *E. coli* DNA polymerase I (New England Biolabs, USA), RNase H (New England Biolabs, USA), and dUTP Solution (Thermo Fisher, USA). Dual‐index adapters were ligated to the fragments, and size selection was performed with AMPureXP beads. After the heat‐labile UDG enzyme (New England Biolabs, USA) treatment of the U‐labelled second‐stranded DNAs, the ligated products were amplified with PCR by under the following conditions: initial denaturation at 95°C for 3 min; 8 cycles of denaturation at 98°C for 15 s, annealing at 60°C for 15 s, and extension at 72°C for 30 s; and then final extension at 72°C for 5 min. The average insert size for the final cDNA libraries was 300 ± 50 bp. Sequencing was performed using 2 × 150 bp paired‐end sequencing (PE150) on an Illumina Novaseq 6000 following the vendor's recommended protocol (Table S2).

RNA‐seq data for male accessory glands, female ovaries, and female heads were generated from a lab‐maintained line (Burns‐Dunn et al. 2024; McKermitt et al. 2024) that originated from the same crickets collected in Las Cruces, 2001 (Ivy and Sakaluk 2005). Approximately 300 adults were housed in ventilated 62.5 L plastic storage bins packed with egg carton. Nymphs were maintained in 6 L bins. Crickets were given *ad libitum* food (approximately equal parts Purina Cat Chow Complete Cat Food and Teklad Global 18% Protein Extruded Rodent Diet pellets) and water (glass vials plugged with moist cotton). Colonies were maintained at a constant temperature (30°C ± 2°C) and under a reversed photoperiod (14:10 h light: dark cycle). Seven‐day‐old crickets were mated, and 2 h later, they were stunned on ice for tissue dissections. Each tissue was immediately snap‐frozen in liquid nitrogen and stored at −80°C until further processing. RNA extractions were performed as described in Foquet et al. (2023). RNA was extracted using a Trizol (Thermo Fisher Scientific) and 1‐bromo‐3‐chloropropane (BCP, Acros Organics) phase separation using standard procedures, followed by a DNAse treatment using a TURBO DNA‐free kit (Thermo Fisher Scientific). RNA qualities and concentrations were measured on a MultiSkan GO microplate spectrophotometer with a μDrop adapter plate (ThermoScientific). Only RNA with final 260/230 and 260/280 values higher than two were used for RNA sequencing. RNA samples were sent to Novogene for library preparation, sequencing, and read pre‐processing. Sample quality was assessed with a Bioanalyzer 2100 (Agilent, USA), with samples obtaining RIN numbers > 5.0 used for sequencing. RNA was processed with an Illumina TruSeq Stranded mRNA Kit with polyA enrichment, and sequencing of paired‐end 150 bp reads on a NovaSeq was performed by Novogene.

### 2.3 Genome Assembly

We used HiFiasm v0.19.5‐r587 (Cheng et al. 2021), with default settings to generate a draft assembly of *G. sigillatus* . The primary assembly was retained for subsequent refinement and analysis. To eliminate duplicates within the contig‐level assembly, Purge_Dups v1.2.6 (Guan et al. 2020) was employed. This identified redundant contigs by mapping the original HiFi reads back to the assembly, removing repetitive or duplicated sequences that could inflate assembly size and misrepresent genomic content. To remove mitochondrial sequences from the draft assembly, MitoHiFi v3.2.1 (Uliano‐Silva et al. 2023) was used. This tool identified and removed contigs associated with the mitochondrial genome by comparing them against the published *G. sigillatus* mitochondrial sequence (MW365703.1, Yang et al. 2021), ensuring that only nuclear genomic sequences remained in the primary assembly.

Hi‐C data were then incorporated to scaffold the contigs and achieve chromosome‐level assembly following Arima Hi‐C mapping guidelines (https://github.com/ArimaGenomics/mapping_pipeline). Briefly, this involved mapping with BWA‐MEM2 v2.0pre2 (Vasimuddin et al. 2019) and removing PCR duplicates using Picard v3.1.0 (https://broadinstitute.github.io/picard). All Hi‐C reads were mapped to the contig‐level assembly, providing spatial genomic interaction information. YahS v1.1a‐r3 (Zhou et al. 2023) was used to scaffold contigs based on these mapping results, integrating long‐range contact information to order and orient contigs accurately. To further refine scaffolding, we used Juicer tools (https://github.com/aidenlab/Juicebox) and Juicebox v3.0.0 (https://github.com/aidenlab/JuicerTools) to visualise the Hi‐C contact heatmap and then manually inspect and correct misassembled regions.

Once the final scaffolds were generated, BlobTools2 (Challis et al. 2020) was used to screen for potential contaminant sequences originating from other species. This step identified 23 scaffolds that likely represented contamination, and these were removed to produce the final, cleaned scaffold‐level genome assembly. This comprehensive, iterative workflow maximised the likelihood of obtaining an accurate, high‐quality chromosome‐level *G. sigillatus* genome assembly suitable for further genomic analyses and annotation.

### 2.4 Genome Quality Assessment

Genome assembly statistics, including total length, N50, and GC content, were calculated using SeqKit v2.4.0 (Shen et al. 2016) to provide a comprehensive overview of the assembly quality. To assess the completeness of the assembled genome, BUSCO v5.4.6 (Manni et al. 2021) was employed, aligning the nucleotide sequences against the Arthropoda dataset (Odb10). This analysis enabled the evaluation of the assembly's coverage and accuracy based on the presence of conserved single‐copy orthologs representative of the Arthropoda lineage.

To evaluate the integrity and accuracy of the genome assembly, we employed the k‐mer‐based method Merqury v1.3 (Rhie et al. 2020). This approach involves mapping k‐mers derived from raw HiFi reads to the contig‐level assembly, enabling a detailed assessment of assembly quality. Specifically, Merqury calculates the Quality Value (QV) score, which serves as a metric for the overall accuracy of the assembly and estimates the error rate at the level of individual bases.

For evaluating the completeness of the protein‐coding gene annotation (see below), BUSCO was again utilised with the Insecta dataset in ‘transcriptome’ mode, which is designed to analyse transcriptome assemblies. This allowed for a thorough assessment of gene prediction quality, ensuring that the annotated protein‐coding genes were representative and complete. By using both genome and transcriptome completeness assessments, we ensured a robust evaluation of the assembly's accuracy and the integrity of its functional annotations.

### 2.5 Genome Annotation

To identify repetitive elements in the genome sequence, three sequential methods were employed to ensure thorough coverage. First, Tandem Repeats Finder v4.09 (TRF; Benson 1999) was used to detect simple sequence repeats. The repetitive regions identified by TRF were masked with ‘N’ in the genome sequence, effectively obscuring these regions for downstream analyses. Next, transposable elements (TEs) within the genome were identified using RepeatMasker v4.1.2‐p1 (Tarailo‐Graovac and Chen 2009), with the previously masked genome sequence serving as input. During this step, the genomic sequences were aligned against the Repbase database v20181026 (Bao et al. 2015) to specifically search for TEs homologous to the Arthropoda lineage. The genome was again subjected to hard‐masking based on the identified TEs. Lastly, RepeatModeler2 (Flynn et al. 2020) was applied to conduct a self‐alignment of the genomic sequences to uncover any additional repetitive elements. The results from RepeatModeler2 were incorporated into a custom repeat database, and RepeatMasker was run once more to identify repeats based on alignment hits. All repetitive elements identified through this comprehensive protocol were soft‐masked in subsequent analyses, preserving their sequence while distinguishing them for further study.

For gene prediction, we integrated both homologous protein sequences and RNA‐seq data. Protein sequences from *D. melanogaster* (BDGP6.46, Ensembl; Harrison et al. 2024), *G. bimaculatus* (Ylla et al. 2021), *T. occipitalis* (Kataoka et al. 2020) and *T. oceanicus* v2 (Zhang et al. 2024), and reviewed orthologs from Arthropoda were obtained from SwissProt to guide the annotation process. Additionally, RNA‐seq data from multiple tissues and both sexes were utilised to facilitate transcriptome‐based annotation. Prior to gene prediction, raw RNA‐seq reads were quality‐trimmed using Fastp v0.23.2 (Chen et al. 2018) and subsequently mapped to the reference genome with HISAT2 v2.2.0 (Kim et al. 2015), ensuring accurate alignment for transcript assembly.

To construct the final gene set, we employed a combined annotation strategy that integrated results from three distinct gene prediction approaches. First, BRAKER3 v3.0 (Gabriel et al. 2024) was used for ab initio gene prediction, leveraging both protein and transcript evidence to train and predict gene structures. Second, Miniprot v0.10‐r225 (Li 2023) was employed to map homologous protein sequences from closely related species and Arthropoda orthologs to the reference genome, providing potential coordinates and open reading frame (ORF) structures of protein‐coding genes. Third, RNA‐seq data were assembled using two different tools, StringTie v2.2.1 (Shumate et al. 2022) and Trinity v2.14.0 (Grabherr et al. 2011), to capture transcript diversity. The Trinity‐assembled transcriptome was filtered to retain sequences longer than 1 Kb, and redundancy was reduced using CD‐HIT v4.8.1 (Li and Godzik 2006). The PASA pipeline v2.5.3 (https://github.com/PASApipeline/PASApipeline) was then utilised to predict gene structures by mapping these transcripts to the reference genome. For the transcriptome assembled by StringTie, potential ORFs were identified using TransDecoder v5.7.1 (https://github.com/TransDecoder/TransDecoder), and coordinates were mapped to the reference genome. Finally, the EvidenceModeler v2.1.0 (Haas et al. 2008) was employed to integrate all prediction results into a unified gene set, while the PASA pipeline refined the final gene models by aligning the transcriptome to predicted gene structures. This comprehensive approach resulted in a high‐confidence, annotated gene set for the target genome.

### 2.6 Functional Annotation

The functions of protein‐coding genes were annotated by mapping the isoform sequences against multiple publicly available functional databases. For Non‐redundant (Sayers et al. 2022) and Swiss‐Prot/TrEMBL (Boeckmann et al. 2003), protein sequences were mapped to an Insecta‐specific subset using DIAMOND blastp v2.0.14.152 (Buchfink et al. 2021), enabling accurate identification of protein functions within this taxonomic group. To identify and annotate functional domains within the protein sequences, InterProScan (Paysan‐Lafosse et al. 2023) was employed, which integrates multiple signature databases for a thorough domain analysis. Additionally, EggNOG mapper (Cantalapiedra et al. 2021) was utilised to conduct EggNOG (Huerta‐Cepas et al. 2019) analysis, from which Gene Ontology (GO) terms were extracted to provide insights into the biological processes, molecular functions, and cellular components associated with each protein. Finally, KEGG pathway (Kanehisa et al. 2016) annotations were acquired using the KAAS online server (Moriya et al. 2007), allowing for the identification of biochemical pathways and the functional context of the proteins within broader metabolic networks.

### 2.7 Ortholog Group Identification and Phylogenetic Analysis

Orthologous protein sequences from six other genomes, including *Anabrus simplex* (NCBI RefSeq accession: GCF_040414725.1 by United States Department of Agriculture), *Gryllus longicercus* (Szrajer et al. 2024), *Gryllus bimaculatus* (Ylla et al. 2021), *Teleogryllus oceanicus* (Zhang et al. 2024), *Teleogryllus occipitalis* (Kataoka et al. 2020) and *Schistocerca americana* (iqSchAmer2.1, https://www.dnazoo.org/assemblies/schistocerca_americana), along with our *G. sigillatus* proteins, were collected for ortholog group identification using OrthoFinder v2.2.5 (Emms and Kelly 2019). To construct a robust phylogenetic framework, we focused on single‐copy orthologs, which were used to build a maximum‐likelihood phylogenetic tree, providing insights into the evolutionary relationships among these species. To estimate divergence times, we incorporated reference divergence times from fossil evidence obtained from the TimeTree database (Kumar et al. 2022), ensuring that our analysis was anchored to well‐established evolutionary benchmarks. The Bayesian inference tool MCMCTree, part of the PAML v4.10.7 package (Reis & Reis and Yang 2011), was then employed to estimate divergence times across all nodes within the phylogeny using only four‐fold sites within all single‐copy gene sequences.

### 2.8 Gene Family Analysis

Using the ultrametric tree generated by MCMCTree, we analysed the expanded and contracted gene families across all nodes of the phylogeny with CAFE5 v1.1 (Mendes et al. 2021), a software designed for modelling gene family evolution. To optimise the analysis, we tested a range of discrete gamma rate (*k*) values from 2 to 6, which account for the variation in evolutionary rates across gene families. The *k* = 4 that yielded the highest maximum‐likelihood was selected for the final model, ensuring an accurate fit to the data. To identify gene families that experienced significant changes specifically in *G. sigillatus* , we conducted a Likelihood Ratio Test (LRT), which enabled us to distinguish gene families that had undergone statistically significant expansions or contractions.

### 2.9 Statistical Analysis and Visualisation

Statistical analyses were performed using R v4.0.2. Diagrams were generated using ggplot2 in the tidyverse package (Wickham et al. 2019). The genomic circos plot was generated using shinyCircos v2.0 (Wang et al. 2023).

## 3 Results and Discussion

### 3.1 Chromosome‐Level Genome Assembly of *G. sigillatus*

We generated a high‐quality, chromosome‐level annotated reference genome for *G. sigillatus* (Figure 1b; Table 1; Table S3). The completed assembly has a total size of 2.17 Gb and comprises 11 pseudochromosomes (Figure 1c; Table S4), consistent with the known karyotype of *G. sigillatus* (You et al. 2007). Based on the latter karyotype study, the longest scaffold in the assembly was marked as chromosome X, and autosomes were named by order of length. The assembly has a scaffold N50 of 200.35 Mb and a maximum scaffold length of 420.78 Mb (Figure 1b, Table S3), and k‐mer based quality estimation yielded a QV score of 61.21 and per‐base error rate of 7.56 × 10^−7^ (Table S4), indicating a highly contiguous and complete genome assembly. To further assess the completeness and quality of the assembly, we performed Benchmarking Universal Single‐Copy Orthologs (BUSCO) analysis using the insecta_odb10, which yielded a BUSCO recovery score of 99.49% (Table S5).

**TABLE 1:** Summary of *Gryllodes sigillatus* genome assembly and annotation.

Genome assembly	*G. sigillatus*
Total length (bp)	2,173,690,933
Contig N50 (bp)	9,022,296
Scaffold N50 (bp)	200,356,516
Longest scaffold (bp)	420,784,874
Hi‐C anchoring rate (%)	98.86
BUSCO (%)	99.49
Repeat content (%)	48.22
Gene number	16,179

Genome annotation revealed that repetitive sequences accounted for 48.22% of the genome, including DNA transposons (16.22%), long interspersed nuclear elements (LINEs, 24.13%), short interspersed nuclear elements (SINEs, 1.81%), and long terminal repeat (LTR) retrotransposons (0.07%) (Figure 1b; Table S6). This repeat content is comparable to that observed in related species (Szrajer et al. 2024; Ylla et al. 2021; Zhang et al. 2024), suggesting a relatively conserved genomic architecture and evolutionary patterns of transposable elements.

We predicted a total of 16,179 protein‐coding genes using a combination of ab initio prediction and evidence‐based methods, incorporating RNA‐seq data (Figure 1b; Table S2) and protein homology. Functional annotation was successful for 84.56% of the total predicted genes, using alignment against several public databases including the Non‐redundant Protein Sequence Database (Nr), TrEMBL, Gene Ontology (GO), EggNOG, and the Kyoto Encyclopedia of Genes and Genomes (KEGG).

### 3.2 Genome Evolution and Phylogenetic Analysis

**FIGURE 2:**
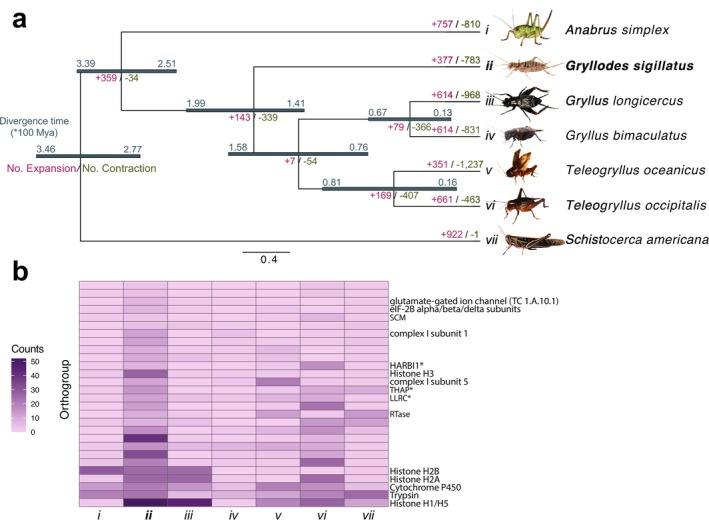
Evolution of gene family expansion and contraction in selected orthopterans. (a) Phylogenetic tree illustrates the divergence times and gene family evolution across seven Orthoptera species. Branch lengths represent evolutionary distances, with upper and lower divergence time estimates (in units of 100 million years ago, Mya) labelled along each branch. Numbers in red and green indicate the number of significantly expanded (+) and contracted (−) gene families along each lineage, respectively. (b) Heatmap illustrating counts of gene families that are significantly expanded in *G. sigillatus* (*ii*), with darker shades representing higher counts. Gene families marked with an asterisk (*) represent those that failed to match a verified family name across Insecta but have homologous sequences in human gene families, indicating possible highly conserved functions. Photo credits: *Anabrus simplex* , *Gryllodes sigillatus* , *Gryllus bimaculatus* and *Teleogryllus oceanicus* by NW Bailey; *Gryllus longicercus* by Jeff Hollenbeck, modified by Shangzhe Zhang, licenced under CC BY‐ND‐NC 1.0; *Teleogryllus occipitalis* by Takaaki Hattori, modified by Shangzhe Zhang, licenced under CC‐BY 4.0; *Schistocerca americana* by Brandon Woo with permission granted.

Our study provides the first reference genome within the *Gryllodes* genus. We also performed a phylogenetic analysis that broadly supports the known evolutionary history of *Gryllodes* (Song et al. 2020). A total of six Ensiferan and one Caeliferan genome (the latter as an outgroup) were selected to identify 15,123 orthogroups, of which 4157 comprised single‐copy orthologs (Figure S1). A maximum‐likelihood species tree was constructed using concatenated alignments of these orthologs, confirming previously reconstructed evolutionary relationships (Song et al. 2020), validating the inferred speciation timeline, and positioning *Gryllodes* within its evolutionary context (Figure 2a). Divergence time estimates indicate that *Gryllodes* diverged from other *Gryllidae* species approximately 141–199 million years ago (Mya).

Analysis of gene family evolution across orthopterans revealed 377 expanded and 783 contracted gene families in *G. sigillatus* (Figure 2a). Focusing on gene families that exhibited significant changes, we identified 28 that were notably expanded in *G. sigillatus* relative to other orthopteran species (Likelihood Ratio Test, *p* < 0.01), consisting of 146 protein‐coding genes in *G. sigillatus* (Table S7). Many of these expanded families are linked to histone‐related functions. Histones are central to nucleosome formation, which packages DNA within the nucleus and regulates gene expression through chromatin remodelling. In insects, histone modifications influence development (Glastad et al. 2019), stress responses (Gupta and Nair 2025), and genome stability (Glastad et al. 2019), and the expansion of histone‐related gene families in *G. sigillatus* may be associated with an enhanced capacity for chromatin remodelling and genome organisation, consistent with adaptations that enable *G. sigillatus* to be resilient in diverse ecological niches.

Other expanded gene families are associated with metabolism and energy production, including those encoding succinyl‐CoA ligase (SCL), NADH dehydrogenase (complex I subunit 1), and trypsin. Succinyl‐CoA ligase, a key enzyme in the tricarboxylic acid (TCA) cycle, catalyses the conversion of succinyl‐CoA to succinate, producing ATP or GTP through substrate‐level phosphorylation. An expansion in SCL genes may enhance TCA cycle efficiency, boosting energy production to meet increased metabolic demands for activities such as locomotion, growth, and reproduction; NADH dehydrogenase, a mitochondrial complex I component, initiates the electron transport chain by transferring electrons from NADH to ubiquinone, driving ATP synthesis via oxidative phosphorylation (Garcia et al. 2017). The expansion of genes encoding NADH dehydrogenase subunits could increase electron transport efficiency, improving ATP generation. Energy metabolism pathways and their evolution in other systems have been linked to the immunity–metabolism interface (Dolezal et al. 2019), detoxification of and resistance to xenobiotics (Gospodaryov et al. 2020), biological invasion (Wang et al. 2013), dispersal and flight (Yang et al. 2014; Treidel et al. 2023). While flight performance is less relevant in *G. sigillatus* , flight muscles are used for male reproductive signalling—acoustic advertisement song—which has been shown to be energetically constrained (Houslay et al. 2017). Overall, metabolic enhancement may further provide *G. sigillatus* with greater resilience to environmental fluctuations in energy availability, supporting sustained activity and survival across diverse conditions. Concentrating on these gene families during selective breeding efforts, for example through marker‐assisted selection, could facilitate genetic improvements in nutritional requirements, energy efficiency, growth rates, and reproductive success.

The expansion of the trypsin gene family suggests adaptations in digestive processes. Trypsins are serine proteases involved in protein digestion in the insect midgut, hydrolysing peptide bonds to aid nutrient absorption (Meriño‐Cabrera et al. 2022; Muhlia‐Almazán et al. 2008). An increased repertoire of trypsin genes may allow *G. sigillatus* to more effectively exploit diverse dietary proteins, supporting rapid growth and high reproductive output. Trypsins are also implicated in immune responses, such as activating the prophenoloxidase system involved in melanisation and pathogen defence (González‐Santoyo and Córdoba‐Aguilar 2012; Kanost and Gorman 2008); thus, their expansion may also enhance immune capabilities in *G. sigillatus* . Determining whether such genes involving immunity are associated with increased resistance to pests and pathogens in a farming setting can help identify strains with specific haplotypes that strengthen immunity to pests, reduce mortality rates, and minimise reliance on chemical treatments. An accurate assessment of genetic variation at such loci across the global range of farmed cricket species is a priority for future work, as the evolutionary potential for responses to artificial selection is currently unknown, and natural variation is likely to be considerably higher than that available in farmed stock populations.

Finally, the expansion of the cytochrome P450 (CYP) gene family, known for its roles in detoxification and the immune response, was observed (Feyereisen 2012). Cytochrome P450 enzymes are involved in the metabolism of endogenous substrates and detoxification of xenobiotics (Nauen et al. 2022), including plant secondary metabolites and synthetic insecticides (Dermauw et al. 2020). Additionally, CYP enzymes contribute to the synthesis and degradation of hormones such as ecdysteroids and juvenile hormones, which regulate development and reproduction. CYP expansion suggests that *G. sigillatus* may possess an enhanced ability to detoxify a broad range of harmful compounds, offering a selective advantage in chemically diverse environments and in energy conversion from agricultural by‐products, thus contributing to more sustainable and economical cricket farming.

These findings highlight potentially distinctive genetics underlying adaptive traits in *G. sigillatus* , shedding light on its evolutionary trajectory within Orthoptera. The expansion of gene families related to chromatin remodelling, metabolism, digestion, and detoxification suggests that *G. sigillatus* has evolved genetic adaptations enhancing its physiological functions and ecological resilience, which are consistent with its global, cosmopolitan distribution in tropical regions and introduction into many non‐native habitats. These adaptations likely support rapid responses to environmental stresses, optimise energy use, and strengthen defences against pathogens and toxins. Genomic resources will help to devise strategies for selectively breeding *G. sigillatus* for optimal reproductive traits in farming applications, as well as for unpicking the evolutionary causes and consequences of nuptial gift‐giving, multiple mating, and mating system evolution in fundamental behavioural ecology research.

## 4 Conclusion

There are over 6000 species of crickets in the infraorder Gryllidea, over 60 of which are known to be consumed and, in some cases, farmed by humans (Cigliano et al. 2018; Magara et al. 2021). Generating a high‐quality chromosome‐scale assembly of one of the most globally widespread and commonly farmed species, *Gryllodes sigillatus* , is a key step in promoting the adoption of genome‐informed farming improvements for insects. The widespread farming of *G. sigillatus* is likely driven by its worldwide, cosmopolitan distribution and ease of collection (Weissman et al. 2012). However, there are many cricket species with farming potential, and the resources developed here will facilitate their study and use more broadly (Dossey et al. 2023; Magara et al. 2019, 2021). Recently, genomic resources, such as genome assemblies and transcriptome sequences, have increasingly been deployed to improve strategies for farming crickets (Kataoka et al. 2022; Nakamura et al. 2022). Comparative and phylogenetic approaches continue to advance our understanding of the evolutionary history of crickets and allied taxa (Gray et al. 2020; Song et al. 2020; Dong et al. 2024), and genomic approaches in these species benefit from a longstanding history of major contributions to fundamental research in evolutionary and behavioural biology. Recent work, for example, has shed light on the genomics of speciation (Xu and Shaw 2021; Yusuf et al. 2024), adaptation in the wild (Pascoal et al. 2020; Zhang et al. 2021, 2024; Rayner et al. 2024), evolutionary developmental biology (Barnett et al. 2019; Ohde et al. 2022), and sperm competition, mate choice, and sexual selection (Simmons et al. 2013; Xu and Shaw 2019). In addition, our findings provide valuable resources for precisely identifying genetic markers using approaches such as genome‐wide association study (GWAS) and marker‐assisted selection, and for guiding the genomic editing of breeding traits through RNA*i* or CRISPR‐Cas9. These technologies have been successfully applied in other farmed crickets, including *Acheta domesticus* (Dossey et al. 2023) and *Gryllus bimaculatus* (Watanabe et al. 2017), and could be similarly implemented in *G. sigillatus* . Future research efforts will benefit not only from exploring the potential for *G. sigillatus* in greater depth using these genome‐informed tools for applied and basic research, but also from developing genomic resources across a broader taxonomic sampling of commonly studied Gryllids.

## Author Contributions

**Shangzhe Zhang:** data curation (equal), formal analysis (lead), methodology (equal), resources (equal), visualization (lead), writing – original draft (equal), writing – review and editing (equal). **Kristin R. Duffield:** data curation (equal), formal analysis (equal), funding acquisition (equal), methodology (equal), resources (equal), writing – review and editing (equal). **Bert Foquet:** data curation (equal), formal analysis (equal), methodology (equal), resources (equal), writing – review and editing (equal). **Jose L. Ramirez:** funding acquisition (equal), supervision (supporting), writing – review and editing (equal). **Ben M. Sadd:** funding acquisition (equal), supervision (supporting), writing – review and editing (equal). **Scott K. Sakaluk:** funding acquisition (equal), supervision (supporting), writing – review and editing (equal). **John Hunt:** funding acquisition (equal), supervision (supporting), writing – review and editing (equal). **Nathan W. Bailey:** conceptualization (lead), funding acquisition (equal), methodology (supporting), project administration (lead), supervision (lead), writing – original draft (equal), writing – review and editing (lead).

## Conflicts of Interest

The authors declare no conflicts of interest.

## Supporting information


Data S1.

Data S2.

## Data Availability

All genomic data (long‐read sequencing data and Hi‐C sequencing data) have been deposited at European Nucleotide Archive (ENA) under PRJEB84028. Transcriptome data have been deposited at PRJEB85996 and PRJNA1204288. Sequences of the genome assembly is publicly available at ENA under accession GCA_965111905. Annotation files of the genome assembly have been deposited at Zenodo at DOI: 10.5281/zenodo.14617131.

## References

[ece371134-bib-0001] Bao, W. , K. K. Kojima , and O. Kohany . 2015. “Repbase Update, a Database of Repetitive Elements in Eukaryotic Genomes.” Mobile DNA 6, no. 1: 11. 10.1186/s13100-015-0041-9. 26045719 PMC4455052

[ece371134-bib-0002] Barnett, A. A. , T. Nakamura , and C. G. Extavour . 2019. “Hox Genes Limit Germ Cell Formation in the Short Germ Insect Gryllus Bimaculatus.” Proceedings of the National Academy of Sciences 116, no. 33: 16430–16435. 10.1073/pnas.1816024116. PMC6697791 31346080

[ece371134-bib-0003] Bateman, P. W. , and D. N. MacFadyen . 1999. “Mate Guarding in the Cricket *Gryllodes sigillatus*: Influence of Multiple Potential Partners.” Ethology 105, no. 11: 949–957. 10.1046/j.1439-0310.1999.00484.x.

[ece371134-bib-0004] Benson, G. 1999. “Tandem Repeats Finder: A Program to Analyze DNA Sequences.” Nucleic Acids Research 27, no. 2: 573–580. 10.1093/nar/27.2.573. 9862982 PMC148217

[ece371134-bib-0006] Boeckmann, B. , A. Bairoch , R. Apweiler , et al. 2003. “The SWISS‐PROT Protein Knowledgebase and Its Supplement TrEMBL in 2003.” Nucleic Acids Research 31, no. 1: 365–370. 10.1093/nar/gkg095. 12520024 PMC165542

[ece371134-bib-0007] Buchfink, B. , K. Reuter , and H.‐G. Drost . 2021. “Sensitive Protein Alignments at Tree‐Of‐Life Scale Using DIAMOND.” Nature Methods 18, no. 4: 366–368. 10.1038/s41592-021-01101-x. 33828273 PMC8026399

[ece371134-bib-0008] Burns‐Dunn, S. , T. Mortys , C. M. House , et al. 2024. “Sexually Antagonistic Coevolution of the Male Nuptial Gift and Female Feeding Behaviour in Decorated Crickets.” Proceedings of the Royal Society B: Biological Sciences 291, no. 2026: 20240804. 10.1098/rspb.2024.0804. PMC11285378 38955230

[ece371134-bib-0009] Cantalapiedra, C. P. , A. Hernández‐Plaza , I. Letunic , P. Bork , and J. Huerta‐Cepas . 2021. “eggNOG‐Mapper v2: Functional Annotation, Orthology Assignments, and Domain Prediction at the Metagenomic Scale.” Molecular Biology and Evolution 38, no. 12: 5825–5829. 10.1093/molbev/msab293. 34597405 PMC8662613

[ece371134-bib-0010] Challis, R. , E. Richards , J. Rajan , G. Cochrane , and M. Blaxter . 2020. “BlobToolKit—Interactive Quality Assessment of Genome Assemblies.” G3: Genes, Genomes, Genetics 10, no. 4: 1361–1374. 10.1534/g3.119.400908. 32071071 PMC7144090

[ece371134-bib-0011] Champagnon, J. , and R. del Cueva Castillo . 2008. “Female Mate Choice, Calling Song and Genetic Variance in the Cricket, *Gryllodes sigillatus* .” Ethology 114, no. 3: 223–230. 10.1111/j.1439-0310.2007.01470.x.

[ece371134-bib-0012] Chen, S. , Y. Zhou , Y. Chen , and J. Gu . 2018. “Fastp: An Ultra‐Fast All‐In‐One FASTQ Preprocessor.” Bioinformatics 34, no. 17: i884–i890. 10.1093/bioinformatics/bty560. 30423086 PMC6129281

[ece371134-bib-0013] Cheng, H. , G. T. Concepcion , X. Feng , H. Zhang , and H. Li . 2021. “Haplotype‐Resolved De Novo Assembly Using Phased Assembly Graphs With Hifiasm.” Nature Methods 18, no. 2: 170–175. 10.1038/s41592-020-01056-5. 33526886 PMC7961889

[ece371134-bib-0014] Cigliano, M. , H. Braun , D. Eades , and D. Otte . 2018. ““Infraorder Gryllidea”. Orthoptera Species File. Version 5.0/5.0. Facultad de Ciencias Naturales y Museo, Universidad Nacional de La Plata (UNLP).”

[ece371134-bib-0015] Dermauw, W. , T. van Leeuwen , and R. Feyereisen . 2020. “Diversity and Evolution of the P450 Family in Arthropods.” Insect Biochemistry and Molecular Biology 127: 103490. 10.1016/j.ibmb.2020.103490. 33169702

[ece371134-bib-0016] Dolezal, T. , G. Krejcova , A. Bajgar , P. Nedbalova , and P. Strasser . 2019. “Molecular Regulations of Metabolism During Immune Response in Insects.” Insect Biochemistry and Molecular Biology 109: 31–42. 10.1016/j.ibmb.2019.04.005. 30959109

[ece371134-bib-0017] Dong, J. , Y. Liu , M. K. Tan , et al. 2024. “Museomics Allows Comparative Analyses of Mitochondrial Genomes in the Family Gryllidae (Insecta, Orthoptera) and Confirms Its Phylogenetic Relationships.” PeerJ 12: e17734. 10.7717/peerj.17734. 39131617 PMC11317039

[ece371134-bib-0018] Dossey, A. T. , B. Oppert , F.‐C. Chu , et al. 2023. “Genome and Genetic Engineering of the House Cricket (*Acheta domesticus*): A Resource for Sustainable Agriculture.” Biomolecules 13, no. 4: 589. 10.3390/biom13040589. 37189337 PMC10136058

[ece371134-bib-0019] Duffield, K. R. , B. Foquet , J. A. Stasko , et al. 2022. “Induction of Multiple Immune Signaling Pathways in *Gryllodes sigillatus* Crickets During Overt Viral Infections.” Viruses 14, no. 12: 2712. 10.3390/v14122712. 36560716 PMC9786821

[ece371134-bib-0020] Duffield, K. R. , J. Hunt , B. M. Sadd , et al. 2021. “Active and Covert Infections of Cricket Iridovirus and *Acheta domesticus* Densovirus in Reared *Gryllodes sigillatus* Crickets.” Frontiers in Microbiology 12: 780796. 10.3389/fmicb.2021.780796. 34917059 PMC8670987

[ece371134-bib-0021] Emms, D. M. , and S. Kelly . 2019. “OrthoFinder: Phylogenetic Orthology Inference for Comparative Genomics.” Genome Biology 20, no. 1: 238. 10.1186/s13059-019-1832-y. 31727128 PMC6857279

[ece371134-bib-0022] Feyereisen, R. 2012. “8—Insect CYP Genes and P450 Enzymes.” In Insect Molecular Biology and Biochemistry, edited by L. I. Gilbert , 236–316. Academic Press. 10.1016/B978-0-12-384747-8.10008-X.

[ece371134-bib-0023] Flynn, J. M. , R. Hubley , C. Goubert , et al. 2020. “RepeatModeler2 for Automated Genomic Discovery of Transposable Element Families.” Proceedings of the National Academy of Sciences 117, no. 17: 9451–9457. 10.1073/pnas.1921046117. PMC7196820 32300014

[ece371134-bib-0024] Foquet, B. , J. Rapkin , M. D. Sharma , B. M. Sadd , S. K. Sakaluk , and J. Hunt . 2023. “Transcriptomic Responses of Females to Consumption of Nuptial Food Gifts as a Potential Mediator of Sexual Conflict in Decorated Crickets.” Journal of Evolutionary Biology 36, no. 1: 183–194. 10.1111/jeb.14114. 36357978

[ece371134-bib-0025] Gabriel, L. , T. Brůna , K. J. Hoff , et al. 2024. “BRAKER3: Fully Automated Genome Annotation Using RNA‐Seq and Protein Evidence With GeneMark‐ETP, AUGUSTUS, and TSEBRA.” Genome Research 34, no. 5: 769–777. 10.1101/gr.278090.123. 38866550 PMC11216308

[ece371134-bib-0026] Garcia, C. J. , J. Khajeh , E. Coulanges , E. I. Chen , and E. Owusu‐Ansah . 2017. “Regulation of Mitochondrial Complex I Biogenesis in Drosophila Flight Muscles.” Cell Reports 20, no. 1: 264–278. 10.1016/j.celrep.2017.06.015. 28683319 PMC5791762

[ece371134-bib-0027] Glastad, K. M. , B. G. Hunt , and M. A. D. Goodisman . 2019. “Epigenetics in Insects: Genome Regulation and the Generation of Phenotypic Diversity.” Annual Review of Entomology 64: 185–203. 10.1146/annurev-ento-011118-111914. 30285490

[ece371134-bib-0028] González‐Santoyo, I. , and A. Córdoba‐Aguilar . 2012. “Phenoloxidase: A Key Component of the Insect Immune System.” Entomologia Experimentalis et Applicata 142, no. 1: 1–16. 10.1111/j.1570-7458.2011.01187.x.

[ece371134-bib-0029] Gospodaryov, D. V. , O. M. Strilbytska , U. V. Semaniuk , et al. 2020. “Alternative NADH Dehydrogenase Extends Lifespan and Increases Resistance to Xenobiotics in Drosophila.” Biogerontology 21, no. 2: 155–171. 10.1007/s10522-019-09849-8. 31749111 PMC7056681

[ece371134-bib-0030] Grabherr, M. G. , B. J. Haas , M. Yassour , et al. 2011. “Full‐Length Transcriptome Assembly From RNA‐Seq Data Without a Reference Genome.” Nature Biotechnology 29, no. 7: 644–652. 10.1038/nbt.1883. PMC3571712 21572440

[ece371134-bib-0031] Grabowski, N. T. , A. Abdulmawjood , F. Acheuk , et al. 2022. “Review: Insects—A Source of Safe and Sustainable Food?— “Jein” (Yes and No).” Frontiers in Sustainable Food Systems 5: 701797. 10.3389/fsufs.2021.701797.

[ece371134-bib-0032] Gray, D. A. , D. B. Weissman , J. A. Cole , E. M. Lemmon , and A. R. Lemmon . 2020. “Multilocus Phylogeny of Gryllus Field Crickets (Orthoptera: Gryllidae: Gryllinae) Utilizing Anchored Hybrid Enrichment.” Zootaxa 4750, no. 3: zootaxa4750. 10.11646/zootaxa.4750.3.2. 32230457

[ece371134-bib-0033] Guan, D. , S. A. McCarthy , J. Wood , K. Howe , Y. Wang , and R. Durbin . 2020. “Identifying and Removing Haplotypic Duplication in Primary Genome Assemblies.” Bioinformatics 36, no. 9: 2896–2898. 10.1093/bioinformatics/btaa025. 31971576 PMC7203741

[ece371134-bib-0034] Gupta, A. , and S. Nair . 2025. “Epigenetic Processes in Insect Adaptation to Environmental Stress.” Current Opinion in Insect Science 67: 101294. 10.1016/j.cois.2024.101294. 39521342

[ece371134-bib-0035] Haas, B. J. , S. L. Salzberg , W. Zhu , et al. 2008. “Automated Eukaryotic Gene Structure Annotation Using EVidenceModeler and the Program to Assemble Spliced Alignments.” Genome Biology 9, no. 1: R7. 10.1186/gb-2008-9-1-r7. 18190707 PMC2395244

[ece371134-bib-0036] Harrison, P. W. , M. R. Amode , O. Austine‐Orimoloye , et al. 2024. “Ensembl 2024.” Nucleic Acids Research 52, no. D1: D891–D899. 10.1093/nar/gkad1049. 37953337 PMC10767893

[ece371134-bib-0037] Houslay, T. M. , K. F. Houslay , J. Rapkin , J. Hunt , and L. F. Bussière . 2017. “Mating Opportunities and Energetic Constraints Drive Variation in Age‐Dependent Sexual Signalling.” Functional Ecology 31, no. 3: 728–741. 10.1111/1365-2435.12766.

[ece371134-bib-0038] Huerta‐Cepas, J. , D. Szklarczyk , D. Heller , et al. 2019. “eggNOG 5.0: A Hierarchical, Functionally and Phylogenetically Annotated Orthology Resource Based on 5090 Organisms and 2502 Viruses.” Nucleic Acids Research 47, no. D1: D309–D314. 10.1093/nar/gky1085. 30418610 PMC6324079

[ece371134-bib-0039] Ivy, T. M. , and S. K. Sakaluk . 2005. “Polyandry Promotes Enhanced Offspring Survival in Decorated Crickets.” Evolution 59, no. 1: 152–159. 10.1111/j.0014-3820.2005.tb00902.x. 15792235

[ece371134-bib-0040] Johnsson, M. 2023. “Genomics in Animal Breeding From the Perspectives of Matrices and Molecules.” Hereditas 160, no. 1: 20. 10.1186/s41065-023-00285-w. 37149663 PMC10163706

[ece371134-bib-0041] Kanehisa, M. , Y. Sato , M. Kawashima , M. Furumichi , and M. Tanabe . 2016. “KEGG as a Reference Resource for Gene and Protein Annotation.” Nucleic Acids Research 44, no. D1: D457–D462. 10.1093/nar/gkv1070. 26476454 PMC4702792

[ece371134-bib-0042] Kanost, M. R. , and M. J. Gorman . 2008. “4—Phenoloxidases in Insect Immunity.” In Insect Immunology, edited by N. E. Beckage , 69–96. Academic Press. 10.1016/B978-012373976-6.50006-9.

[ece371134-bib-0043] Kasdorf, S. Y. , M. J. Muzzatti , F. Haider , S. M. Bertram , and H. A. MacMillan . 2025. “Brewery Waste as a Sustainable Protein Source for the Banded Cricket ( *Gryllodes sigillatus* ).” Journal of Insects as Food and Feed 1: 1–13. 10.1163/23524588-00001368.

[ece371134-bib-0044] Kataoka, K. , R. Minei , K. Ide , et al. 2020. “The Draft Genome Dataset of the Asian Cricket Teleogryllus Occipitalis for Molecular Research Toward Entomophagy.” Frontiers in Genetics 11: 470. 10.3389/fgene.2020.00470. 32457806 PMC7225344

[ece371134-bib-0045] Kataoka, K. , Y. Togawa , R. Sanno , T. Asahi , and K. Yura . 2022. “Dissecting Cricket Genomes for the Advancement of Entomology and Entomophagy.” Biophysical Reviews 14, no. 1: 75–97. 10.1007/s12551-021-00924-4. 35340598 PMC8921346

[ece371134-bib-0046] Kim, D. , B. Langmead , and S. L. Salzberg . 2015. “HISAT: A Fast Spliced Aligner With Low Memory Requirements.” Nature Methods 12, no. 4: 357–360. 10.1038/nmeth.3317. 25751142 PMC4655817

[ece371134-bib-0047] Kong, J. D. , M. W. Ritchie , É. Vadboncoeur , H. A. MacMillan , and S. M. Bertram . 2024. “Growth, Development, and Life History of a Mass‐Reared Edible Insect, *Gryllodes sigillatus* (Orthoptera: Gryllidae).” bioRxiv: 2024.10.08.617250. 10.1101/2024.10.08.617250. PMC12167847 40251933

[ece371134-bib-0048] Kumar, S. , M. Suleski , J. M. Craig , et al. 2022. “TimeTree 5: An Expanded Resource for Species Divergence Times.” Molecular Biology and Evolution 39, no. 8: msac174. 10.1093/molbev/msac174. 35932227 PMC9400175

[ece371134-bib-0049] Lange, K. W. , and Y. Nakamura . 2023. “Potential Contribution of Edible Insects to Sustainable Consumption and Production.” Frontiers in Sustainability 4: 1112950. 10.3389/frsus.2023.1112950.

[ece371134-bib-0050] Li, H. 2023. “Protein‐To‐Genome Alignment With Miniprot.” Bioinformatics 39, no. 1: btad014. 10.1093/bioinformatics/btad014. 36648328 PMC9869432

[ece371134-bib-0051] Li, W. , and A. Godzik . 2006. “Cd‐Hit: A Fast Program for Clustering and Comparing Large Sets of Protein or Nucleotide Sequences.” Bioinformatics 22, no. 13: 1658–1659. 10.1093/bioinformatics/btl158. 16731699

[ece371134-bib-0052] Magara, H. J. O. , S. Niassy , M. A. Ayieko , et al. 2021. “Edible Crickets (Orthoptera) Around the World: Distribution, Nutritional Value, and Other Benefits—A Review.” Frontiers in Nutrition 7: 537915. 10.3389/fnut.2020.537915. 33511150 PMC7835793

[ece371134-bib-0053] Magara, H. J. O. , C. M. Tanga , M. A. Ayieko , et al. 2019. “Performance of Newly Described Native Edible Cricket Scapsipedus Icipe (Orthoptera: Gryllidae) on Various Diets of Relevance for Farming.” Journal of Economic Entomology 112, no. 2: 653–664. 10.1093/jee/toy397. 30657915

[ece371134-bib-0054] Manni, M. , M. R. Berkeley , M. Seppey , and E. M. Zdobnov . 2021. “BUSCO: Assessing Genomic Data Quality and Beyond.” Current Protocols 1, no. 12: e323. 10.1002/cpz1.323. 34936221

[ece371134-bib-0055] McKermitt, J. T. , B. Foquet , W. Kuna , J. Hunt , B. M. Sadd , and S. K. Sakaluk . 2024. “Experimental Evolution Under Varying Sex Ratio and Behavioral Plasticity in Response to Perceived Competitive Environment Independently Affect Calling Effort in Male Crickets.” Evolution 78, no. 3: 453–462. 10.1093/evolut/qpad224. 38124480

[ece371134-bib-0056] Mendes, F. K. , D. Vanderpool , B. Fulton , and M. W. Hahn . 2021. “CAFE 5 Models Variation in Evolutionary Rates Among Gene Families.” Bioinformatics 36, no. 22–23: 5516–5518. 10.1093/bioinformatics/btaa1022. 33325502

[ece371134-bib-0057] Meriño‐Cabrera, Y. B. , M. G. D. A. Oliveira , Y. B. Meriño‐Cabrera , and M. G. D. A. Oliveira . 2022. “Trypsins: Structural Characterization and Inhibition Focus in Insects.” In Hydrolases. IntechOpen. 10.5772/intechopen.102632.

[ece371134-bib-0058] Morales‐Ramos, J. A. , M. G. Rojas , A. T. Dossey , and M. Berhow . 2020. “Self‐Selection of Food Ingredients and Agricultural By‐Products by the House Cricket, *Acheta domesticus* (Orthoptera: Gryllidae): A Holistic Approach to Develop Optimized Diets.” PLoS One 15, no. 1: e0227400. 10.1371/journal.pone.0227400. 31978186 PMC6980616

[ece371134-bib-0059] Moriya, Y. , M. Itoh , S. Okuda , A. C. Yoshizawa , and M. Kanehisa . 2007. “KAAS: An Automatic Genome Annotation and Pathway Reconstruction Server.” Nucleic Acids Research 35, no. suppl_2: W182–W185. 10.1093/nar/gkm321. 17526522 PMC1933193

[ece371134-bib-0060] Muhlia‐Almazán, A. , A. Sánchez‐Paz , and F. L. García‐Carreño . 2008. “Invertebrate Trypsins: A Review.” Journal of Comparative Physiology B 178, no. 6: 655–672. 10.1007/s00360-008-0263-y. 18404270

[ece371134-bib-0061] Muzzatti, M. J. , J. D. Kong , E. R. McColville , et al. 2025a. “Diet Particle Size Influences Tropical House Cricket Life History.” Journal of Insects as Food and Feed 1: 1–11. 10.1163/23524588-00001365.

[ece371134-bib-0062] Muzzatti, M. J. , M. W. Ritchie , E. C. Bess , S. M. Bertram , and H. A. MacMillan . 2025b. “Farmed Crickets (Orthoptera: Gryllidae) Raised With Dermestids (Coleoptera: Dermestidae) Suffer From Reduced and Delayed Growth, but Not Enough to Explain Reports of Dramatic Yield Loss.” Journal of Economic Entomology 118, no. 1: 160–171. 10.1093/jee/toae208. 39278631 PMC11818372

[ece371134-bib-0063] Nakamura, T. , G. Ylla , and C. G. Extavour . 2022. “Genomics and Genome Editing Techniques of Crickets, an Emerging Model Insect for Biology and Food Science.” Current Opinion in Insect Science 50: 100881. 10.1016/j.cois.2022.100881. 35123119

[ece371134-bib-0064] Nauen, R. , C. Bass , R. Feyereisen , and J. Vontas . 2022. “The Role of Cytochrome P450s in Insect Toxicology and Resistance.” Annual Review of Entomology 67, no. Volume 67, 2022: 105–124. 10.1146/annurev-ento-070621-061328. 34590892

[ece371134-bib-0065] Ohde, T. , T. Mito , and T. Niimi . 2022. “A Hemimetabolous Wing Development Suggests the Wing Origin From Lateral Tergum of a Wingless Ancestor.” Nature Communications 13, no. 1: 979. 10.1038/s41467-022-28624-x. PMC8861169 35190538

[ece371134-bib-0066] Pascoal, S. , J. E. Risse , X. Zhang , et al. 2020. “Field Cricket Genome Reveals the Footprint of Recent, Abrupt Adaptation in the Wild.” Evolution Letters 4, no. 1: 19–33. 10.1002/evl3.148. 32055408 PMC7006468

[ece371134-bib-0067] Paysan‐Lafosse, T. , M. Blum , S. Chuguransky , et al. 2023. “InterPro in 2022.” Nucleic Acids Research 51, no. D1: D418–D427. 10.1093/nar/gkac993. 36350672 PMC9825450

[ece371134-bib-0068] Rapkin, J. , K. Jensen , C. R. Archer , et al. 2018. “The Geometry of Nutrient Space–Based Life‐History Trade‐Offs: Sex‐Specific Effects of Macronutrient Intake on the Trade‐Off Between Encapsulation Ability and Reproductive Effort in Decorated Crickets.” American Naturalist 191, no. 4: 452–474. 10.1086/696147. 29570407

[ece371134-bib-0069] Rayner, J. G. , F. Eichenberger , J. V. A. Bainbridge , et al. 2024. “Competing Adaptations Maintain Nonadaptive Variation in a Wild Cricket Population.” Proceedings of the National Academy of Sciences 121, no. 32: e2317879121. 10.1073/pnas.2317879121. PMC11317585 39088392

[ece371134-bib-0070] Reis, M. D. , and Z. Yang . 2011. “Approximate Likelihood Calculation on a Phylogeny for Bayesian Estimation of Divergence Times.” Molecular Biology and Evolution 28, no. 7: 2161–2172. 10.1093/molbev/msr045. 21310946

[ece371134-bib-0071] Rhie, A. , B. P. Walenz , S. Koren , and A. M. Phillippy . 2020. “Merqury: Reference‐Free Quality, Completeness, and Phasing Assessment for Genome Assemblies.” Genome Biology 21, no. 1: 245. 10.1186/s13059-020-02134-9. 32928274 PMC7488777

[ece371134-bib-0072] Sakaluk, S. K. 1984. “Male Crickets Feed Females to Ensure Complete Sperm Transfer.” Science 223, no. 4636: 609–610. 10.1126/science.223.4636.609. 17749941

[ece371134-bib-0073] Sakaluk, S. K. 1991. “Post‐Copulatory Mate Guarding in Decorated Crickets.” Animal Behaviour 41, no. 2: 207–216. 10.1016/S0003-3472(05)80472-5.

[ece371134-bib-0074] Sakaluk, S. K. , K. R. Duffield , J. Rapkin , B. M. Sadd , and J. Hunt . 2019. “The Troublesome Gift: The Spermatophylax as a Purveyor of Sexual Conflict and Coercion in Crickets.” In Advances in the Study of Behavior, edited by M. Naguib , L. Barrett , S. D. Healy , J. Podos , L. W. Simmons , and M. Zuk , vol. 51, 1–30. Academic Press. 10.1016/bs.asb.2018.12.001.

[ece371134-bib-0075] Sakaluk, S. K. , J. M. Schaus , A. Eggert , W. A. Snedden , and P. L. Brady . 2002. “Polyandry and Fitness of Offspring Reared Under Varying Nutritional Stress in Decorated Crickets.” Evolution 56, no. 10: 1999–2007. 10.1111/j.0014-3820.2002.tb00126.x. 12449487

[ece371134-bib-0076] Sayers, E. W. , E. E. Bolton , J. R. Brister , et al. 2022. “Database Resources of the National Center for Biotechnology Information.” Nucleic Acids Research 50, no. D1: D20–D26. 10.1093/nar/gkab1112. 34850941 PMC8728269

[ece371134-bib-0077] Shen, W. , S. Le , Y. Li , and F. Hu . 2016. “SeqKit: A Cross‐Platform and Ultrafast Toolkit for FASTA/Q File Manipulation.” PLoS One 11, no. 10: e0163962. 10.1371/journal.pone.0163962. 27706213 PMC5051824

[ece371134-bib-0078] Shumate, A. , B. Wong , G. Pertea , and M. Pertea . 2022. “Improved Transcriptome Assembly Using a Hybrid of Long and Short Reads With StringTie.” PLoS Computational Biology 18, no. 6: e1009730. 10.1371/journal.pcbi.1009730. 35648784 PMC9191730

[ece371134-bib-0079] Simmons, L. W. , Y.‐F. Tan , and A. H. Millar . 2013. “Sperm and Seminal Fluid Proteomes of the Field Cricket Eleogryllus Oceanicus: Identification of Novel Proteins Transferred to Females at Mating.” Insect Molecular Biology 22, no. 1: 115–130. 10.1111/imb.12007. 23211034

[ece371134-bib-0080] Sinha, P. , V. K. Singh , A. Bohra , A. Kumar , J. C. Reif , and R. K. Varshney . 2021. “Genomics and Breeding Innovations for Enhancing Genetic Gain for Climate Resilience and Nutrition Traits.” Theoretical and Applied Genetics 134, no. 6: 1829–1843. 10.1007/s00122-021-03847-6. 34014373 PMC8205890

[ece371134-bib-0081] Song, H. , O. Béthoux , S. Shin , et al. 2020. “Phylogenomic Analysis Sheds Light on the Evolutionary Pathways Towards Acoustic Communication in Orthoptera.” Nature Communications 11, no. 1: 4939. 10.1038/s41467-020-18739-4. PMC7532154 33009390

[ece371134-bib-0082] Szrajer, S. , D. Gray , and G. Ylla . 2024. “The Genome Assembly and Annotation of the Cricket Gryllus Longicercus.” Scientific Data 11, no. 1: 708. 10.1038/s41597-024-03554-z. 38942791 PMC11213874

[ece371134-bib-0083] Tarailo‐Graovac, M. , and N. Chen . 2009. “Using RepeatMasker to Identify Repetitive Elements in Genomic Sequences.” Current Protocols in Bioinformatics 5, no. 1: 4–10. 10.1002/0471250953.bi0410s25. 19274634

[ece371134-bib-0084] Treidel, L. A. , P. Goswami , and C. M. Williams . 2023. “Changes in Mitochondrial Function Parallel Life History Transitions Between Flight and Reproduction in Wing Polymorphic Field Crickets.” American Journal of Physiology. Regulatory, Integrative and Comparative Physiology 324, no. 6: R735–R746. 10.1152/ajpregu.00191.2022. 37036301

[ece371134-bib-0085] Uliano‐Silva, M. , J. G. R. N. Ferreira , K. Krasheninnikova , et al. 2023. “MitoHiFi: A Python Pipeline for Mitochondrial Genome Assembly From PacBio High Fidelity Reads.” BMC Bioinformatics 24, no. 1: 288. 10.1186/s12859-023-05385-y. 37464285 PMC10354987

[ece371134-bib-0086] van Huis, A. , and D. G. A. B. Oonincx . 2017. “The Environmental Sustainability of Insects as Food and Feed. A Review.” Agronomy for Sustainable Development 37, no. 5: 43. 10.1007/s13593-017-0452-8.

[ece371134-bib-0088] Vasimuddin, M. , S. Misra , H. Li , and S. Aluru . 2019. “Efficient Architecture‐Aware Acceleration of BWA‐MEM for Multicore Systems.” In International Parallel and Distributed Processing Symposium (IPDPS), 314–324. IEEE. 10.1109/IPDPS.2019.00041.

[ece371134-bib-0089] Wang, Y. , L. Jia , G. Tian , et al. 2023. “shinyCircos‐V2.0: Leveraging the Creation of Circos Plot With Enhanced Usability and Advanced Features.” iMeta 2, no. 2: e109. 10.1002/imt2.109. 38868422 PMC10989951

[ece371134-bib-0090] Wang, Y.‐L. , Y.‐J. Wang , J.‐B. Luan , G.‐H. Yan , S.‐S. Liu , and X.‐W. Wang . 2013. “Analysis of the Transcriptional Differences Between Indigenous and Invasive Whiteflies Reveals Possible Mechanisms of Whitefly Invasion.” PLoS One 8, no. 5: e62176. 10.1371/journal.pone.0062176. 23667457 PMC3648516

[ece371134-bib-0091] Watanabe, T. , S. Noji , and T. Mito . 2017. “Genome Editing in the Cricket, Gryllus Bimaculatus.” Methods in Molecular Biology 1630: 219–233. 10.1007/978-1-4939-7128-2-18. 28643262

[ece371134-bib-0092] Weissman, D. B. , D. A. Gray , H. T. Pham , and P. Tijssen . 2012. “Billions and Billions Sold: Pet‐Feeder Crickets (Orthoptera: Gryllidae), Commercial Cricket Farms, an Epizootic Densovirus, and Government Regulations Make for a Potential Disaster.” Zootaxa 3504, no. 1: 67–88. 10.11646/zootaxa.3504.1.3.

[ece371134-bib-0093] Wickham, H. , M. Averick , J. Bryan , et al. 2019. “Welcome to the Tidyverse.” Journal of Open Source Software 4, no. 43: 1686. 10.21105/joss.01686.

[ece371134-bib-0094] Xu, M. , and K. L. Shaw . 2019. “Genetic Coupling of Signal and Preference Facilitates Sexual Isolation During Rapid Speciation.” Proceedings of the Royal Society of London B 286, no. 1913: 20191607. 10.1098/rspb.2019.1607. PMC6834049 31640515

[ece371134-bib-0095] Xu, M. , and K. L. Shaw . 2021. “Extensive Linkage and Genetic Coupling of Song and Preference Loci Underlying Rapid Speciation in Laupala Crickets.” Journal of Heredity 112, no. 2: 204–213. 10.1093/jhered/esab001. 33438016

[ece371134-bib-0096] Yang, J. , H. Dong , M. He , and J. Gao . 2021. “Mitochondrial Genome Characterization of *Gryllodes sigillatus* (Orthoptera: Gryllidae) and Its Phylogenetic Implications.” Mitochondrial DNA Part B Resources 6, no. 3: 1056–1058. 10.1080/23802359.2021.1899078. 33796736 PMC7995884

[ece371134-bib-0097] Yang, Y. , S. Xu , J. Xu , Y. Guo , and G. Yang . 2014. “Adaptive Evolution of Mitochondrial Energy Metabolism Genes Associated With Increased Energy Demand in Flying Insects.” PLoS One 9, no. 6: e99120. 10.1371/journal.pone.0099120. 24918926 PMC4053383

[ece371134-bib-0098] Ylla, G. , T. Nakamura , T. Itoh , et al. 2021. “Insights Into the Genomic Evolution of Insects From Cricket Genomes.” Communications Biology 4, no. 1: 733. 10.1038/s42003-021-02197-9. 34127782 PMC8203789

[ece371134-bib-0099] You, J.‐X. , N. Li , B.‐Z. Ren , H.‐B. Yuan , and L.‐N. Shang . 2007. “The Study on Karyotypes of Five Grylloidea Species (Orthoptera: Grylloidea) in Northeast China.” Zootaxa 1611, no. 1: 63–68. 10.11646/zootaxa.1611.1.5.

[ece371134-bib-0100] Yusuf, L. H. , S. Pascoal , P. A. Moran , and N. W. Bailey . 2024. “Testing the Genomic Overlap Between Intraspecific Mating Traits and Interspecific Mating Barriers.” Evolution Letters 8, no. 6: 902–915. 10.1093/evlett/qrae042. 39677567 PMC11637687

[ece371134-bib-0101] Zhang, X. , M. Blaxter , J. M. D. Wood , et al. 2024. “Temporal Genomics in Hawaiian Crickets Reveals Compensatory Intragenomic Coadaptation During Adaptive Evolution.” Nature Communications 15, no. 1: 5001. 10.1038/s41467-024-49344-4. PMC11169259 38866741

[ece371134-bib-0102] Zhang, X. , J. G. Rayner , M. Blaxter , and N. W. Bailey . 2021. “Rapid Parallel Adaptation Despite Gene Flow in Silent Crickets.” Nature Communications 12, no. 1: 50. 10.1038/s41467-020-20263-4. PMC7782688 33397914

[ece371134-bib-0103] Zhou, C. , S. A. McCarthy , and R. Durbin . 2023. “YaHS: Yet Another Hi‐C Scaffolding Tool.” Bioinformatics 39, no. 1: btac808. 10.1093/bioinformatics/btac808. 36525368 PMC9848053

