# Supplementary material for: A High‐Quality Reference Genome and Comparative Genomics of the Widely Farmed Banded Cricket ( Gryllodes sigillatus ) Identifies Selective Breeding Targets
Source: Ecol Evol. 2025 Mar 16;15(3):e71134. doi: 10.1002/ece3.71134 (PMC11911027; doi:10.1002/ece3.71134)
Supplement: Supplementary file 2 — Data S2. [file ECE3-15-e71134-s001.pdf]

Supplementary Materials for

A high-quality reference genome and comparative genomics of the widely-farmed banded cricket (*Gryllodes sigillatus*) identifies selective breeding targets

Shangzhe Zhang[1], Kristin R. Duffield[2,3], Bert Foquet[4,5], Jose L. Ramirez[3], Ben M. Sadd[4], Scott K. Sakaluk[4], John Hunt[6], Nathan W. Bailey[1,*]

[1]School of Biology, University of St Andrews, St Andrews, Fife KY16 9TH, UK
[2]USDA-ARS, Geospatial and Environmental Epidemiology Research Unit, Mississippi State, MS, USA
[3]USDA-ARS, National Center for Agricultural Utilization Research, Crop BioProtection Research Unit, Peoria, IL, USA
[4]School of Biological Sciences, Illinois State University, Normal, IL 61790, USA
[5]McGuire Center for Lepidoptera and Biodiversity, Florida Museum of Natural History, University of Florida, Gainesville, FL, USA
[6]School of Science, Western Sydney University, Hawkesbury Campus, Penrith NSW 2751, Australia

* Correspondence to Nathan W. Bailey: nwb3@st-andrews.ac.uk

This supplementary file includes:

Figs. S1
Table S1 to S7

Other Supplementary materials for this manuscript include:

Supplementary data table 1

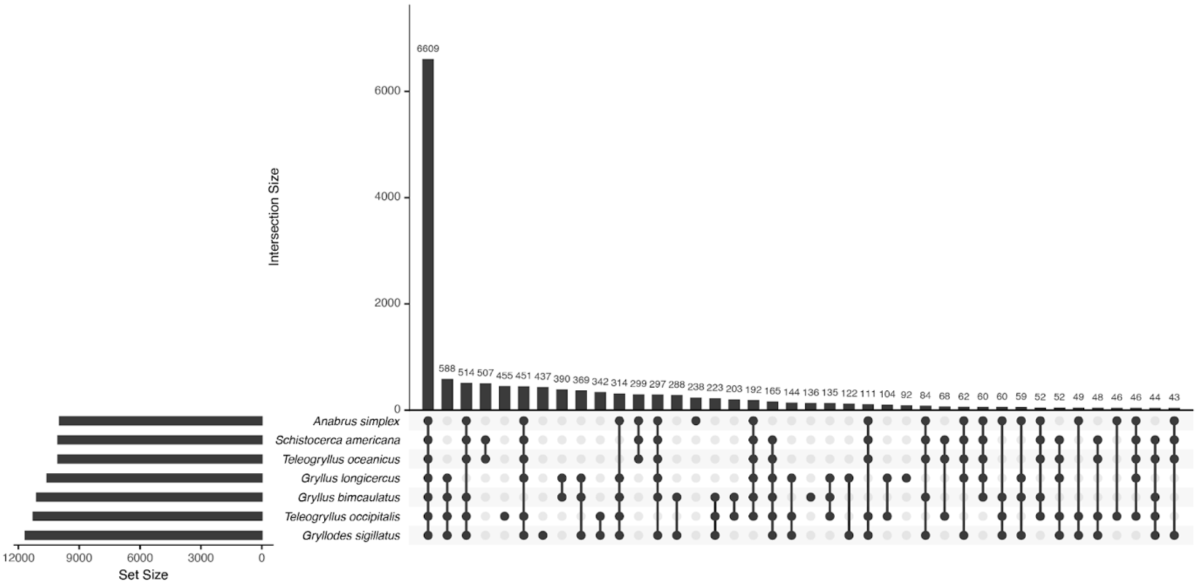

Supplementary figure 1. UpSet plot illustrating the overlap of gene families among seven species. The horizontal bars on the left represent the total number of gene families identified in each species. The vertical bars in the main panel indicate the number of gene families shared among the species combinations connected by the filled dots below.

Supplementary table 1. Summary of the raw sequencing data.

Library Type	Base (Gb)	Average read length (bp)	Coverage Depth (×)
PacBio HiFi	96.25	17,427.4	48.13
Hi-C	151.48	150	72.33

Supplementary table 2. Information of RNA-seq data used for protein-coding gene prediction.

Sample name	Tissue	Sex	Mapping ratio (%)
F3AG10	Accessory Glands	Male	94.58
F3AG2	Accessory Glands	Male	94.64
F3AG8	Accessory Glands	Male	94.76
F3H1	Head	Female	92.99
F3H2	Head	Female	93.47
F3H3	Head	Female	95.14
F3RP1	Ovaries	Female	96.55
F3RP2	Ovaries	Female	96.63
F3RP3	Ovaries	Female	96.36
KD1	Whole body	Female	94.81
KD2	Whole body	Female	94.79
KD3	Whole body	Female	94.58
KD6	Whole body	Male	95.67
KD7	Whole body	Male	96.78
KD8	Whole body	Male	97.82

Supplementary table 3. Statistics of *G. sigillatus* genome assembly.

Statistics	Value
**Total length (bp)**	2,173,690,933
**Contig number**	455
**Contig N50 (Mb)**	9.02
**Scaffold number**	101
**Scaffold N50 (Mb)**	200.36
**GC content (%)**	39.77

Supplementary table 4. Quality assessment of the assembled genome of *G. sigillatus*.

Chromosome name	Scaffold name	Contig number	Length (Mb)	QV	Error rate (×10^{-7} per base)
ChrX	scaffold_1	200	420.78	60.91	8.11
Chr1	scaffold_2	49	243.29	61.71	6.74
Chr2	scaffold_3	39	237.93	62.50	5.62
Chr3	scaffold_4	41	200.36	60.66	8.59
Chr4	scaffold_5	34	197.45	60.39	9.14
Chr5	scaffold_6	50	190.27	62.22	5.99
Chr6	scaffold_7	31	148.10	61.58	6.95
Chr7	scaffold_8	41	143.15	62.18	6.05
Chr8	scaffold_9	43	136.84	60.30	9.33
Chr9	scaffold_10	42	124.84	61.40	7.24
Chr10	scaffold_11	36	124.84	60.12	9.72
**Entire genome**		455	2,173.70	61.21	7.56

Supplementary table 5. Quality assessment of the assembled genome and gene set of *G. sigillatus* using BUSCOs. The reference BUSCO set was insecta_odb10 with a total BUSCO number of 1367.

Object	Type	Number	Percent (%)
Assembly	Complete BUSCOs (C)	1360	**99.5**
	Complete and single-copy BUSCOs (S)	1342	98.2
	Complete and duplicated BUSCOs (D)	18	1.3
	Fragmented BUSCOs (F)	0	0
	Missing BUSCOs (M)	7	0.5
Gene set	Complete BUSCOs (C)	1347	**98.5**
	Complete and single-copy BUSCOs (S)	916	67.0
	Complete and duplicated BUSCOs (D)	431	31.5
	Fragmented BUSCOs (F)	4	0.3
	Missing BUSCOs (M)	16	1.2

Supplementary table 6. Summary of repeat elements in *G. sigillatus* genome.

Repeats type	Number	Length (bp)	Percentage of Repeats (%)	Percentage of Genome (%)
**Transposable elements**	-	996,365,344	94.90	45.77
**SINEs**	68,919	19,030,453	1.81	0.87
**LINEs**	400,691	253,317,115	24.13	12.64
**LTRs**	110,496	731,976	0.070	3.30
**DNAs**	413,510	170,341,526	16.22	7.82
**Rolling circles**	65,312	25,082,240	2.39	1.15
**Unclassified**	1,638,266	479,417,892	45.66	22.03
**Small RNA**	38,674	13,708,717	13.06	0.64
**Satellites**	613	173,884	0.017	0.01
**Simple repeats**	445,329	22,189,720	2.11	1.02
**Low complexity**	85,828	69,333	-	0.22
**Total Repeats**	-	1,049,877,180	-	48.22

Supplementary table 7. Details of significantly expanded protein-coding gene families in *G. sigillatus*. Gene families marked with an asterisk (*) represent those that failed to have a verified family name across Insecta but have homologous sequences in human gene families, indicating possible conserved functions across species.

Ortholog identifier	Gene family name	Gene number in *G. sigillatus*	P value
OG0000001	Histone H1/H5	52	2.88e-10
OG0000005	Trypsin	23	0.0028
OG0000009	Cytochrome P450	21	0.040
OG0000021	Histone H2A	26	1.16e-05
OG0000022	Histone H2B	25	2.62e-05
OG0000055	*Unknown*	20	1.8e-07
OG0000060	*Unknown*	33	3.32e-15
OG0000061	*Unknown*	16	0.00088
OG0000065	*Unknown*	41	5.16e-23
OG0000080	*Unknown*	17	8.25e-06
OG0000104	*Unknown*	8	0.013
OG0000106	RTase	9	0.0053
OG0000117	*Unknown*	13	2.16e-05
OG0000123	LLRC*	12	0.0044
OG0000124	THAP*	15	1.47e-06
OG0000125	Complex I subunit 5	11	0.00029
OG0000146	Histone H3	27	2.22e-15
OG0000197	HARBI1*	8	0.0018
OG0000418	*Unknown*	10	0.00013
OG0000450	*Unknown*	9	4.07e-05
OG0000453	*Unknown*	12	1.36e-08
OG0000494	Complex I subunit 1	12	4.81e-07
OG0000772	*Unknown*	5	0.012
OG0000886	SCM	3	0.020
OG0000941	eIF-2B alpha/beta/delta subunits	7	3.66e-05
OG0001510	Glutamate-gated ion channel	7	3.66e-05
OG0001843	*Unknown*	4	0.0042
OG0007868	*Unknown*	3	0.020
